# Supplementary material for: Content-rich biological network constructed by mining PubMed abstracts
Source: BMC Bioinformatics. 2004 Oct 8;5:147. doi: 10.1186/1471-2105-5-147 (PMC528731; doi:10.1186/1471-2105-5-147)
Supplement: Additional File 2 — The original results of the above study (non-essential files are deleted to keep the file size under the limit set by BMC bioinformatics). [file 1471-2105-5-147-S2.bz2 › chilibotAdditionalFile2/dip05/580ID8624812E10948/html/HLA_B2M.html]

 


 **HLA** and **B2M** 
  
Found 815 abstracts in PubMed, retrieved 05.  
 

 What does Google say? 
 PDF only 
| .edu only 

---

**Interactive relationship** (e.g. stimulation, inhibition, etc)

**Non-interactive relationship** (e.g. studied together, co-existance, homology, etc.)

- The regions analyzed consisted of the  **HLA**  class I heavy chain genes located within the MHC genomic region on chromosome arm 6p, the  **HLA**  class I light chain beta 2 microglobulin  [ **B2M** ] ,  **B2M**  gene on chromosome arm 15q, and the putative  **HLA**  modifier of methylation gene MEMO1 located on chromosome arm 1q.  Ref: 12759931 Genes Chromosomes Cancer, 2003
- These cells were systematically characterized by Northern and or Western blotting e.g., mini transcriptome mini proteome analysis for the expression of  **HLA**  A, B, C, beta 2 microglobulin  [ **B2M** ] , and the members of the antigen processing machinery of class I molecules LMP2, LMP7, TAP1, TAP2, tapasin, calreticulin, calnexin, and ERp57.  Ref: 12874016 Cancer Res, 2003
- In contrast to the high frequency of APM component down regulation,  **HLA**  class I heavy chain and beta 2 microglobulin  [ **B2M** ]  defects were detected in only 12 and 10% of the lesions, respectively.  Ref: 12738726 Clin Cancer Res, 2003
